# Spontaneous Regression of Advanced Transverse Colon Cancer: A Case Report

**DOI:** 10.70352/scrj.cr.24-0018

**Published:** 2025-01-31

**Authors:** Shinya Ohno, Yoshinori Iwata, Saki Mitsutome, Shusaku Kawai, Manabu Neo, Moe Fukuda, Bei Wang, Tomonari Suetsugu, Taku Watanabe, Shuji Komori, Chihiro Tanaka, Narutoshi Nagao, Masahiko Kawai

**Affiliations:** Department of Surgery, Gifu Prefectural General Medical Center, Gifu, Gifu, Japan

**Keywords:** spontaneous regression, high-frequency microsatellite instability, deficient mismatch repair

## Abstract

**INTRODUCTION:**

The incidence of spontaneous regression (SR) of malignancy is one in 60000–100000 cancer patients and spontaneous regression in colorectal cancer is quite rare, reported to account for less than 2% of spontaneous regression of malignancy. In recent years, some reports of spontaneous regression in colorectal cancer in patients with high-frequency microsatellite instability have suggested a deep association between high-frequency microsatellite instability and spontaneous regression. We report our experience of spontaneous regression of advanced colorectal cancer with high-frequency microsatellite instability and provide a review of spontaneous regression in colorectal cancer.

**CASE PRESENTATION:**

An 83-year-old woman was diagnosed as having advanced colorectal cancer in the transverse colon by lower gastrointestinal endoscopy, and biopsy results revealed moderately differentiated adenocarcinoma. Contrast-enhanced computed tomography showed a tumor located near the hepatic flexure and an enlarged lymph node near the tumor. No distant metastasis was observed, and the preoperative diagnosis was cT3N1aM0 cStage IIIb cancer. Immunohistochemical analysis of the biopsy specimen suggested deficient mismatch repair. During the wait for surgery, the patient was urgently hospitalized due to severe dehydration. After her general condition improved, 38 days after the biopsy, we performed laparoscopic resection of the partial ascending and transverse colon with D3 lymph node dissection. The tumor noted preoperatively was not present in the specimen, and intraoperative endoscopy revealed no tumor on the anorectal side. Additional ileocecal resection was performed, but no tumor was found in the specimen, and another intraoperative endoscopy was performed, which revealed a discolored scar near the anal margin. We determined that tumor loss or morphological change had occurred, so after additional resection of the same area, ultimately, an extended right hemicolectomy was performed. Histopathological diagnosis was pT0N0M0 pStage0 cancer with no residual tumor. The patient has progressed without recurrence at 1 year after the operation.

**CONCLUSIONS:**

The immunological response due to high-frequency microsatellite instability may be related to the mechanism of spontaneous regression in colorectal cancer. If high-frequency microsatellite instability is diagnosed preoperatively, we recommend that the tumor location should be confirmed preoperatively.

## Abbreviations

SR
: spontaneous regression
CRC
: colorectal cancer
MSI-H
: high-frequency microsatellite instability
dMMR
: deficient mismatch repair
POD
: postoperative day

## INTRODUCTION

Spontaneous regression (SR) of malignancy is defined as the partial or total disappearance of a histologically diagnosed malignant primary or disseminated lesion with no treatment or with treatment considered insufficient to have a significant impact on tumorigenic disease.^1,2)^ SR has been reported in all cancer types^3)^ but mainly in renal cell carcinoma, malignant lymphoma, leukemia, neuroblastoma, and malignant melanoma.^4–10)^ The overall estimated incidence is one in 60000–100000 cancer patients,^11)^ and SR in colorectal cancer (CRC) is relatively rare, reported to account for less than 2% of SR of malignancy.^12)^ In recent years, scattered reports of SR of CRC in patients with high-frequency microsatellite instability (MSI-H) have suggested a possible link between MSI-H and SR.^13,14)^ We report our experience of SR of advanced CRC with MSI-H and review the reports of SR in CRC.

## CASE PRESENTATION

**Fig. 1:**
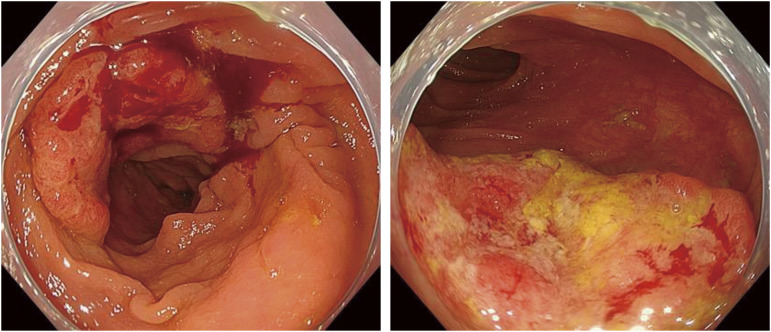
Lower endoscopy image. A type 2 advanced colon cancer of 30 mm in size with moderately differentiated adenocarcinoma is shown in the transverse colon.

An 83-year-old woman with postprandial nausea and epigastric pain was brought to her previous hospital and then referred to our Department of Gastroenterology for cholecystolithiasis and acute cholecystitis. After conservative treatment, she underwent a lower gastrointestinal endoscopy as preoperative evaluation for cholecystectomy, which revealed a type 2 advanced CRC of 30 mm in size with moderately differentiated adenocarcinoma in the transverse colon (**Fig. 1**).

**Fig. 2:**
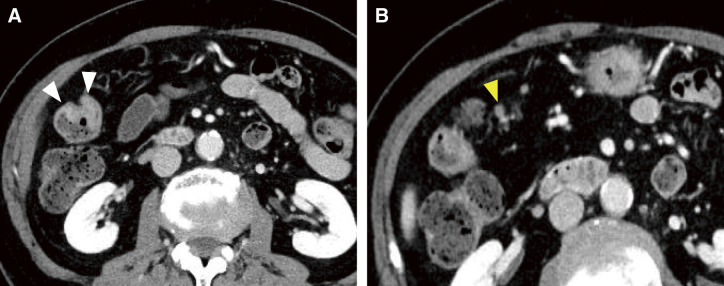
Contrast-enhanced computed tomography images. Images show a tumor located near the hepatic flexure (white arrowheads) (**A**) and an enlarged lymph node near the tumor (yellow arrowhead) (**B**).

**Fig. 3:**
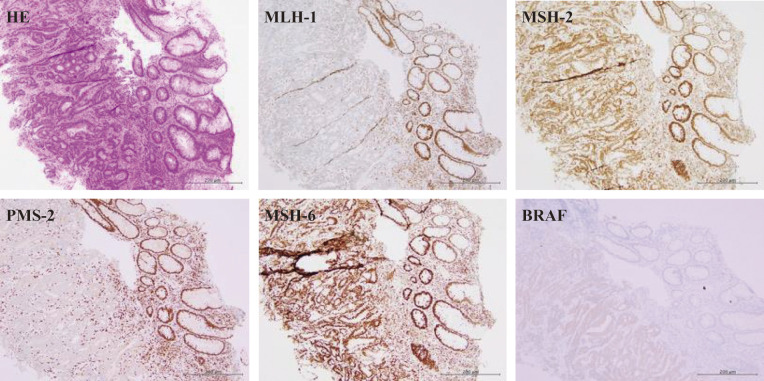
Immunohistochemical analysis. Immunohistochemical analysis of biopsy specimens showed that MLH-1 and PMS-2 were not expressed and MutS homolog 2 (MSH-2) and MSH-6 were remained, suggesting deficient mismatch repair, and BRAF V600E was a mutant type. MLH-1, MutL homolog 1; PMS-2, postmeiotic segregation increased 2

**Fig. 4:**
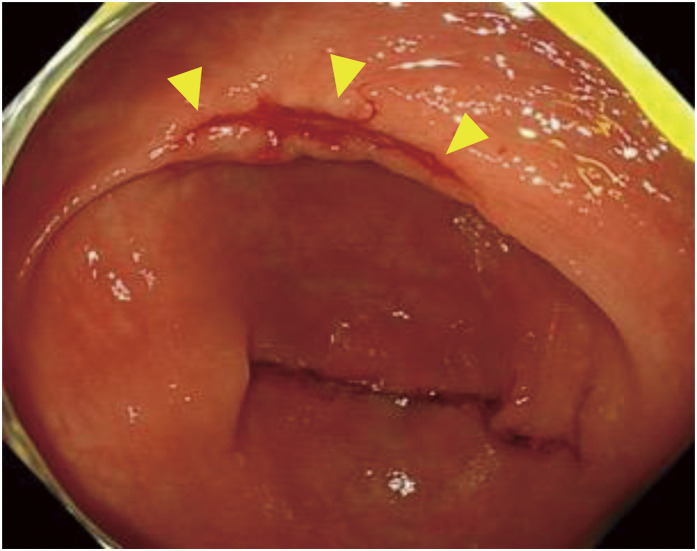
Intraoperative lower endoscopic image. Intraoperative lower endoscopy showed a discolored scar near the anal margin (yellow arrowheads).

**Fig. 5:**
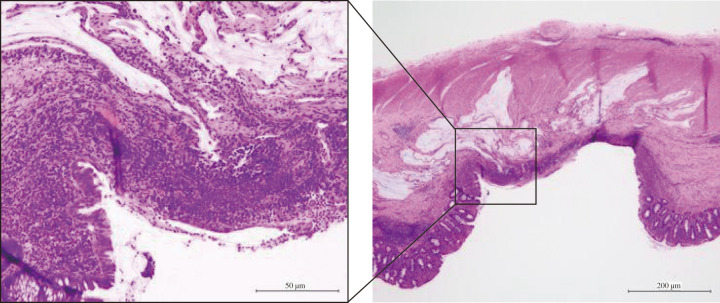
Histopathological image. Staining shows pT0N0M0 pStage0 with no residual tumor and granular stromal growth with inflammatory cell infiltration in the surrounding area (inset).

The patient’s height was 160 cm, weight was 52.6 kg, and body mass index was 20.5 kg/m^2^. At her initial visit to our department, her performance status was 1, and there were no abnormal physical findings of note. Blood tests showed liver and renal function to be within normal limits, and there was no anemia or elevated tumor markers, although there was a mildly elevated inflammatory response due to the cholecystitis. Contrast-enhanced computed tomography showed a tumor located near the hepatic flexure and an enlarged lymph node near the tumor (**Fig. 2**). No distant metastasis was observed, and the preoperative diagnosis was cT3N1aM0 cStageIIIb. Immunohistochemical analysis of the biopsy specimens showed that MutL homolog 1 and postmeiotic segregation increased 2 were not expressed, suggesting deficient mismatch repair (dMMR) (**Fig. 3**). RAS was of the wild type, and BRAF V600E was a mutant type. During the waiting period for surgery, the patient was admitted to our emergency room with anorexia and difficulty moving and was urgently admitted due to dehydration. After her general condition improved, 38 days after the biopsy, we performed laparoscopic resection of the partial ascending and transverse colon with D3 lymph node dissection. However, the tumor noted preoperatively was not present in the specimen, and intraoperative endoscopy revealed no tumor on the anorectal side. After determining that the tumor remained on the oral side, an additional ileocecal resection was performed. However, no tumor was found in this specimen, and intraoperative endoscopy was performed again, which revealed a discolored scar at the residual transverse colon near the resection stump (**Fig. 4**). Although the morphology of the tumor differed significantly from that noted preoperatively, there were no other obvious abnormalities on the mucosal surface, and it was determined that tumor loss or morphological change had occurred. So after additional resection of the remaining transverse colon, ultimately, an extended right hemicolectomy was performed. The operative time was 459 min, and the amount of blood loss was 210 mL. The histopathological diagnosis was pT0N0M0 pStage0 with no residual tumor and granular stromal growth with inflammatory cell infiltration in and around the submucosa and muscularis propria of the ulcerous lesion (**Fig. 5**). The patient started oral intake on postoperative day (POD) 3. Her oral intake decreased due to loss of appetite, and she required supplemental fluids on POD 7, but there were no other complications. She was discharged on POD 16. The patient has progressed without recurrence at 1 year after the operation.

## Discussion

The occurrence of SR in CRC is exceedingly rare, likely due to prompt surgical intervention typically initiated upon diagnosis.^5)^ Previous reports have described a variety of potential factors contributing to SR, including prolonged fever associated with sepsis, physical stimuli such as biopsy, primary tumor resection, formation of stoma, and psychological and genetic influences.^12)^ However, the precise underlying mechanisms remain elusive.

Proposed mechanisms for SR in CRC include ischemia by tumor growth, mechanical stimulation by intestinal peristalsis, circulatory failure by tumor torsion or traction, physical stimulation from biopsy, and an immune response characterized by the generation of specific antibodies against tumor antigens.^1,15)^ The high frequency of SR in renal cell carcinoma and malignant melanoma with high levels of neoantigen suggests the possibility of an antitumor immune response as the mechanism of SR. Chida et al.^16)^ reported SR might have been attributable to an immune-mediated anti-tumor response mediated primarily by CD4+ T cells in biopsy specimens. Recent reports on SR in CRC have also revealed an association with dMMR, suggesting the possibility of SR mediated by antitumor immune responses.

Abdelrazeq et al.^12)^ summarized 21 cases of SR in CRC between 1900 and 2005, and up to 2024, 44 cases in total have been reported. Among the 22 cases reported since 2000, which include only those with primary tumor or regional lymph node metastasis, including our case, the median age was 75 (60–86) years, and 50% were female^12–29)^ (**Table 1**). The primary tumor locations were cecum (n = 3), ascending (n = 6) and transverse (n = 10) colon, and rectum (n = 2). Cancer was found in the right colon in 19 cases (90.5%), more frequently than in the left colon and rectum, suggesting an association with MSI-H.^12^) Tumor morphology was mostly type 0–II or type 2, median tumor size was 20 (8–30) mm, and histology was tub1 (n = 7), tub2 (n = 10), and por (n = 4). Our case and only one other case were suspected of having lymph node metastasis before surgery.^25)^ The median duration of tumor disappearance from initial diagnosis was 2 (1–7) months. SR has often been considered to be an immunologic mechanism, and the fact that all reported cases in which MMR states were examined showed dMMR suggests that an immunogenic mechanism is involved.^13,14,25–27)^

**Table 1:** Reported cases of spontaneous regression of colorectal cancer

Author	Year	Age	Sex	Primary site	Type	Size (mm)	Histology	Clinical diagnosis	Operation	MMR states	Proposed reason	Duration (months)	Follow-up (years)
Kamesui	2000	66	F	Ascending	0-I sp	20	tub2	T1N0M0	+	ND	Dislodged	2	1
Tomiki	2007	80	F	Rectum	0-IIa+IIc	20	tub1	T1N0M0	–	ND	Dislodged	ND	5
Sakamoto	2009	80	M	Rectum	1	25	tub1	T2N0M0	+	ND	Not reported	3	5
Shimizu	2010	80	M	Transverse	2	25	tub2	T2N0M0	–	ND	Dislodged	7	1.5
Nakashima	2012	76	F	Cecum	0-Ip	20	tub1	T1N0M0	+	ND	Dislodged	2	5
Sekiguchi	2013	69	F	Ascending	0-IIa	20	tub2	T1N0M0	+	ND	Immunologic	1.5	1.5
Nakamura	2013	60	M	Rectum	0-IIa+IIc	10	tub1	T1N0M0	–	ND	Biopsy	1	ND
Serizawa	2015	75	M	Transverse	0-IIc+IIa	15	tub1	T1N0M0	–	ND	Not clear	2.5	1
Kihara	2015	64	M	Transverse	2	30	tub2	T2N0M0	+	ND	Immunologic	1.5	1
Chida	2017	80	M	Transverse	2	30	por	T2N0M0	+	ND	Immunologic	1	1
Karakuchi	2019	70	M	Transverse	2	30	por	T2N0M0	+	dMMR	Immunologic	2	5
Nishiura	2020	67	F	Transverse	2	13	por	T2N1aM0	+	dMMR	Immunologic	3	1.2
Utsumi	2021	78	M	Ascending	0-IIa+IIc	8	tub1	T1N0M0	+	dMMR	Immunologic	1	1
Utsumi	2021	66	M	Ascending	0-IIa+IIc	10	tub2	T1N0M0	+	dMMR	Immunologic	1.5	1.5
Utsumi	2021	73	M	Ascending	0-IIa+IIc	10	tub2	T1N0M0	+	dMMR	Immunologic	1	5.5
Yokota	2021	76	F	Transverse	0-Is+IIc	10	tub2	T1N0M0	+	dMMR	Immunologic	2	5
Yokota	2021	64	F	Cecum	0-Is	15	tub1	T1N0M0	+	dMMR	Immunologic	3	6
Yokota	2021	64	M	Transverse	2	20	tub2	T2N0M0	+	dMMR	Immunologic	1.5	6
Harata	2023	76	F	Transverse	2	30	tub1	T2N0M0	+	dMMR	Immunologic	2	ND
Shuttleworth	2023	78	F	Ascending	1	22	por	T2N0M0	+	ND	Immunologic and inflammatory or dislodged	5	3.5
Shuttleworth	2023	86	F	Cecum	0-IIc+IIa		tub2	T1N0M0	+	ND	Immunologic and inflammatory	1	1
Our case	2024	83	F	Transverse	2	30	tub2	T3N1aM0	+	dMMR	Immunologic and severe debilitation; dehydration	1	1

MMR, mismatch repair; ND, not described; dMMR, deficient mismatch repair

It has been reported that a marked infiltration of CD4+ T cells is observed in CRCs of MSI-H lacking MHC class II expression,^29)^ and CD4+ T cells are present along the cancer stroma in the SR portion in CRC, suggesting an adaptive immunological response to cancer.^16)^ Nishiura et al.^25)^ reported that the primary tumor was SR, but the regional metastatic lymph nodes neither disappeared nor shrank, suggesting that the gene expression profile of metastatic cancer may be different from that of primary cancer. The mechanism of SR could be one of the direct physical stimulation of the tumor by the biopsy eliciting an immunological response that caused SR only in the primary tumor. In our case, in addition to biopsy stimulation, the preoperative exposure to the physical stress of severe dehydration may have activated an immunological response that led to SR.

Although there have been no reports of recurrence, there have been cases in which lymph node metastases have not disappeared, even if the primary site of CRC has completely disappeared,^16)^ and cases in which tumor components have remained in the resection specimen.^28)^

In our case, the tumor was judged to be advanced and its location could be identified intraoperatively, so surgery was performed without tattooing or clipping near the tumor before surgery. A discolored scar was found at the residual transverse colon near the resection stump on intraoperative endoscopy, but we did not expect the tumor to have disappeared, and this resulted in an enlarged resection that we regret. If the tumor is not detected intraoperatively despite the obvious presence of tumor formation in the preoperative diagnosis, the possibility of SR should be considered, and lower endoscopy should be performed with attention to scar tissue as well. Also, if MSI-H is diagnosed preoperatively and there is a delay between the time of initial diagnosis and surgery, it may be necessary to reconfirm the location of the tumor by lower endoscopy immediately before surgery. Even if SR was confirmed preoperatively, we consider that tattooing or clipping should be performed to locate the tumor preoperatively.

In other reports from Japan, the incidence of MSI-H was 4.5%–10% in stage II or III CRC cases, and the incidence of dMMR was 13.3% in patients with stage II CRC and 9.1% in stage III CRC.^30–32)^ However, many of the patients with SR of CRC were diagnosed as having dMMR, suggesting that dMMR CRC is more likely to cause SR than CRC with proficient MMR, although it is unclear whether only CRC with dMMR causes SR. It was reported that the BRAF V600E mutation rate in stage II or III dMMR CRCs was 35.3%,^33)^ whereas the BRAF mutation rate was 68.3% and 60.7% among patients with dMMR stage II and III CRC in our hospital.^34)^ The BRAF V600E variant is also found in 35%–43% of patients with MSI-H CRC,^35)^ CRC diagnosed as sporadic MSI-H is not uncommon. Our patient had the BRAF V600E variant, which aided in the diagnosis of sporadic MSI-H. The frequency of SR may differ depending on BRAF status; however, the details are not clear because BRAF status has not been reported in previous cases. It is also possible that the frequency of SR differs between sporadic MSI-H CRC and Lynch syndrome.

## CONCLUSIONS

The immunological response due to MSI-H may be related to the mechanism of SR in CRC. If MSI-H is diagnosed preoperatively and the waiting time for surgery is prolonged, preoperative endoscopy and marking near the tumor should be performed before surgery, and the possibility of tumor disappearance should be considered.

## ACKNOWLEDGMENTS

We thank Rise Japan LLC (http://rise-japan.rulez.jp/) for editing the English language of this manuscript.

## DECLARATIONS

### Funding

Not applicable.

### Authors’ contributions

SO drafted the manuscript.

SM, YI, and CT performed the operation.

YI, SKa, MN, MF, BW, TS, TW, SKo, NN, and MK provided academic advice.

All authors have read and approved the final manuscript and agree to be responsible for all aspects of the study.

### Availability of data and materials

Not applicable.

### Ethics approval and consent to participate

This report was approved by the institutional review board of Gifu Prefectural General Medical Center (approval no. 969).

### Consent for publication

Not applicable.

### Competing interests

The authors certify that they have no affiliations with or involvement in any organization or entity with any financial interest (such as honoraria; educational grants; participation in speakers’ bureaus; membership, employment, consultancies, stock ownership, or other equity interest; or expert testimony or patent-licensing arrangements), or non-financial interest (such as personal or professional relationships, affiliations, knowledge, or beliefs) in the subject matter or materials discussed in this manuscript.
